# Disparities in COVID-19 vaccine intentions, testing and trusted sources by household language for children with medical complexity

**DOI:** 10.1371/journal.pone.0305553

**Published:** 2024-06-14

**Authors:** Laura P. Chen, Kristina Singh-Verdeflor, Michelle M. Kelly, Daniel J. Sklansky, Kristin A. Shadman, M. Bruce Edmonson, Qianqian Zhao, Gregory P. DeMuri, Ryan J. Coller

**Affiliations:** 1 Department of Pediatrics, School of Medicine and Public Health, University of Wisconsin-Madison, Madison, Wisconsin, United States of America; 2 Department of Biostatistics and Medical Informatics, School of Medicine and Public Health, University of Wisconsin-Madison, Madison, Wisconsin, United States of America; BOKU: Universitat fur Bodenkultur Wien, AUSTRIA

## Abstract

**Objectives:**

Children with medical complexity experienced health disparities during the coronavirus disease 2019 (COVID-19) pandemic. Language may compound these disparities since people speaking languages other than English (LOE) also experienced worse COVID-19 outcomes. Our objective was to investigate associations between household language for children with medical complexity and caregiver COVID-19 vaccine intentions, testing knowledge, and trusted sources of information.

**Methods:**

This cross-sectional survey of caregivers of children with medical complexity ages 5 to 17 years was conducted from April-June 2022. Children with medical complexity had at least 1 Complex Chronic Condition. Households were considered LOE if they reported speaking any language other than English. Multivariable logistic regression examined associations between LOE and COVID-19 vaccine intentions, interpretation of COVID-19 test results, and trusted sources of information.

**Results:**

We included 1,338 caregivers of children with medical complexity (49% response rate), of which 133 (10%) had household LOE (31 total languages, 58% being Spanish). There was no association between household LOE and caregiver COVID-19 vaccine intentions. Caregivers in households with LOE had similar interpretations of positive COVID-19 test results, but significantly different interpretations of negative results. Odds of interpreting a negative test as expected (meaning the child does not have COVID-19 now or can still get the virus from others) were lower in LOE households (aOR [95% CI]: 0.56 [0.34–0.95]). Households with LOE were more likely to report trusting the US government to provide COVID-19 information (aOR [95% CI]: 1.86 [1.24–2.81]).

**Conclusion:**

Differences in COVID-19 test interpretations based on household language for children with medical complexity were observed and could contribute to disparities in outcomes. Opportunities for more inclusive public health messaging likely exist.

## Introduction

The COVID-19 pandemic has reinforced many healthcare disparities in the US [1]. Rates of COVID-19 vaccination, infection, hospitalization, and mortality illustrate stark inequities across racially and ethnically diverse populations [2–6], as well as those with chronic conditions [7–11]. Unfortunately, language disparities have further exacerbated inequities. Some patients who speak a language other than English (LOE) [12] have lower vaccine intentions, higher proportion of COVID-19 disease incidence, and a higher mortality rate; though these relationships likely vary across populations speaking different LOE [13–17].

While children have generally experienced less severe COVID-19 health consequences than adults [18], some pediatric groups, such as children with medical complexity (CMC) [19] are at greater risk. CMC are at high risk of both infection and poor outcomes from COVID-19 [20–24]. Early in the pandemic, CMC had 8 times higher risk of hospitalization from COVID-19 and comprised a high proportion of patients admitted to pediatric intensive care units [8, 25]. Since parent language has been associated with poor health care outcomes, such as lack of insurance and decreased health care access for children with special health care needs [26–29], it is possible that household language may worsen existing COVID-19 health disparities for CMC.

The purpose of this study was to investigate the association between CMC household language and caregiver perceptions around COVID-19 vaccination, testing, and trusted information sources. Our hypothesis was that in the CMC population, LOE status may promote disparities in COVID-19-related perceptions that may influence their health outcomes. Different perceptions based on household language may have important implications for designing public health interventions related to COVID-19 and other viral respiratory illnesses for CMC.

## Methods

### Study design, setting, and participants

This was a cross-sectional analysis of survey data from caregivers of CMC (“caregivers”) recruited from a large Midwestern United States academic medical center from April 1, 2022 to June 30, 2022. The survey was conducted as part of the National Institutes of Health’s Rapid Acceleration of Diagnostics-Underserved Populations (RADx-UP) program, which aims “to ensure that all Americans have access to COVID-19 testing, with a focus on communities most affected by the pandemic” [30]. This study reports data from the most recent of three data collection periods, following the Strengthening the Reporting of Observational Studies in Epidemiology (STROBE) reporting statement. Eligible participants were identified via the electronic medical record based on the following criteria: at least 18 years of age and cared for CMC between 5–17 years old, attended school before March 2020, had at least 2 encounters at our medical center in 2020, at least 1 complex chronic condition (CCC), and had an address in the electronic medical record. CCCs are any medical condition that can be reasonably expected to last at least 12 months (unless death intervenes) and involve several different organ systems or 1 organ system severely enough to require specialty care and probable hospitalization at a tertiary care center [31].

Individuals were invited to participate in this survey via mailing coordinated by our university survey center. Eligible caregivers received a $5 incentive with each survey; caregivers that completed surveys received $50. Up to 3 surveys were mailed to each participant. Caregivers that participated in prior data collection periods could elect to receive the survey by email. Surveys were available in English and Spanish; participants whose children were identified in the electronic medical record as Spanish-speaking were mailed a survey in Spanish. This study was approved by the University of Wisconsin-Madison’s institutional review board (#2021–0515) and participant written informed consent was obtained electronically or by mail. The survey was comprised of questions from the RADx-UP common data elements library [30], a bank of publicly available, standardized questions including respondent and family demographics, social determinants of health, medical history, and COVID-19 perceptions and practices (including vaccination, testing, and trusted sources of information).

### Outcomes, exposure, and covariates

The first dependent variable of interest was COVID-19 vaccine intentions, defined dichotomously as present if caregivers responded “yes” to “Has your child received a COVID-19 vaccine?” or responded “very likely” to “How likely is your child to get an approved COVID-19 vaccine when it becomes available?” Vaccine intention was defined as absent if caregivers responded “no” to “Has your child received a COVID-19 vaccine?” and responded anything other than “very likely” to “How likely is your child to get an approved COVID-19 vaccine when it becomes available?”

The second dependent variable of interest was COVID-19 test result interpretation, defined as present if caregivers responded as expected to two scenarios: “If my child gets a positive test result…” and “If my child gets a negative test result…”. Expected response for a positive test result was choosing both 1) “My child will need to isolate themselves from others” and 2) “My child will not be able to attend school”. Expected response for a negative test result was choosing “My child doesn’t have COVID-19 now” and not choosing “My child can be around others without getting the virus from them.” The RADx-UP common data elements test perceptions questions do not distinguish between specific tests such a rapid antigen or polymerase chain reaction.

Caregiver sources of trust for pandemic information were assessed as secondary outcomes to guide potential public health messaging strategies that might best influence primary outcomes. Trust was defined as present if caregivers responded “a great deal” to the question, “How much do you trust each of these sources to provide correct information about COVID-19 as it relates to your child?” Information sources included your doctor or health care provider, faith leader, close friends and members of your family, people you go to work or class with or people you know, your contacts on social media, the US government, and the US Coronavirus Task Force. Trust was defined as absent if caregivers responded, “not at all”, “a little”, “somewhat” or “don’t know”.

The exposure of interest was LOE status. Households were defined as having LOE if they answered “yes” to “Do you speak a language other than English at home?” Covariates were modeled categorically and included child’s age, health insurance, number of CCCs (1 vs ≥2), hospital encounters in the year before enrollment (0 vs ≥1), caregiver’s highest level of education, annual household income, and perceived severity of COVID-19 for their child. In the primary outcome analysis, we included sources of trust for COVID-19 information as a covariate.

### Analysis

Participant demographic and clinical characteristics are summarized by descriptive statistics. Bivariable followed by multivariable logistic regression models estimated associations between the dependent variable, exposure of interest, and covariates. After confirming no collinearity (all Variance Inflation Factors were <1.5) and model stability, covariates with statistically significant bivariable associations with the primary outcomes were included in multivariable models. We also included *a priori* selected covariates that were suggested by prior published research as potential confounders, regardless of statistical significance in the bivariable models [32, 33]. Due to the small sample size, we did not evaluate for interactions. To explore the potential influence of response bias, a sensitivity analysis was conducted using Chi-squared tests to assess differences between respondents and nonrespondents, using available data from the creation of the eligible cohort (S1 Table). Using these variables, we also created propensity scores to assess the probability of survey response using logistic regression models [34]. The primary analysis was repeated with inverse probability weighting by applying these propensity scores.

Sample size was estimated from the parent study [35], where n = 900 would have adequate power to detect moderate effect sizes (1.5 ≤ OR ≤ 2.0) at the two-sided 0.05 significance level in cross-sectional logistic regression models predicting binary outcomes with 80% power under various scenarios and binary predictors (x = 0- absent/x = 1- present). Analyses were conducted in SASv.9.4. P-values <0.05 were considered statistically significant.

## Results

### Participant characteristics and household language

Among 2,897 mailed surveys, 1,432 caregivers returned surveys (49% response rate) and 1,338 (46%) responded to the outcome questions (Table 1). Regarding household language, 133 (10%) of respondents reported LOE, of which 58% identified Spanish, 8% Mandarin, and 7% American Sign Language, in addition to 28 other unique languages.

**Table 1:** Demographic and clinical characteristics of caregivers and children overall and by language other than English (LOE) status.

	All n (col %)	LOE n (col %)	Not LOE n (col %)
Total, *n (row %)*	1338 (100)	133 (10)	1205 (90)
**Caregiver Characteristics**			
Age			
Median (IQR)	43 (39–48)	43 (37–50)	43 (39–47)
Caregiver Race and Ethnicity			
White, Non-Hispanic	1174 (88)	51 (38)	1123 (93)
Hispanic	71 (5)	52 (39)	19 (2)
Black, Non-Hispanic	22 (2)	6 (5)	16 (1)
Other race, Non-Hispanic	44 (3)	21 (16)	23 (2)
Multiracial, Non-Hispanic	14 (1)	2 (1)	12 (1)
Not reported	13 (1)	1 (1)	12 (1)
Primary Language			
English	1205 (90)	0	1205 (100)
Spanish	77 (6)	77 (58)	0
Mandarin	11 (1)	11 (8)	0
American Sign Language	9 (1)	9 (7)	0
Other	36 (2)	36 (27)	0
Highest Education			
< 12th Grade	29 (2)	16 (12)	13 (1)
GED or Some College	480 (36)	47 (36)	433 (36)
Bachelor’s Degree	470 (35)	31 (23)	439 (36)
Advanced Degree	359 (27)	39 (29)	320 (27)
Income			
< $35,000	150 (11)	23 (17)	127 (11)
$35,000–$49,999	94 (7)	15 (11)	79 (7)
$50,000–$74,999	182 (14)	22 (17)	160 (13)
$75,000–$99,999	199 (15)	16 (12)	183 (15)
> $100,000	567 (42)	34 (26)	533 (44)
Not Reported	146 (11)	23 (17)	123 (10)
Primary Insurance Type^1^			
Public Insurances	486 (36)	62 (47)	424 (35)
Private Insurances	821 (61)	60 (45)	761 (63)
Other Insurances	31 (3)	11 (8)	20 (2)
**Child Characteristics**			
Sex			
Male	702 (52)	72 (54)	630 (52)
Female	629 (47)	59 (44)	570 (47)
Other	7 (1)	2 (2)	5 (1)
Age			
< 12 years old	540 (40)	60 (45)	480 (40)
≥ 12 years old	798 (60)	73 (55)	725 (60)
Number of Complex Chronic Conditions			
1	948 (71)	100 (75)	848 (70)
2 or more	390 (29)	33 (25)	357 (30)
Hospital Admissions in 2020			
0	1095 (82)	108 (81)	987 (82)
1 or more	243 (18)	25 (19)	218 (18)
Ever Tested Positive for COVID-19			
Yes	456 (34)	51 (38)	405 (34)
No	787 (59)	74 (56)	713 (59)
Missing	95 (7)	8 (6)	87 (7)

^1^The ’Public Insurances’ category includes participants with both public and private insurance. The ‘Other Insurances’ category includes participants who selected “Does not have health insurance”, “Don’t Know”, or “Prefer not to answer.”

Child characteristics in households with and without LOE were similar, including child sex, age, number of CCCs, hospital encounters in the prior year, and prior positive COVID-19 test. Just over one-quarter of CMC had ≥2 CCCs and nearly 20% had hospital encounters in 2020.

Caregiver characteristics differed more between households with and without LOE. For example, households reporting LOE had a greater proportion of caregivers with lower education level, lower income and public insurance.

Respondents and non-respondents had similar child characteristics for age, sex, and number of complex chronic conditions. However, a higher proportion of respondents identified as non-Hispanic white and utilized private insurance as compared to non-respondents (S1 Table).

### Primary outcomes and LOE status: COVID-19 vaccine intentions and testing

No significant associations between household LOE and COVID-19 vaccine intentions were observed (adjusted odds ratio, aOR [95%CI]: 1.28 [0.74–2.23] between LOE and non-LOE groups) (Table 2). COVID-19 vaccine intentions were 80% in both households with and without LOE (Table 3). Households with LOE were significantly less likely to interpret negative COVID-19 test results as expected (aOR [95% CI]: 0.56 [0.34–0.95]). Specifically, households with LOE were significantly more likely to report that a negative test means that their child can be around others without getting the virus from them (P<0.0001, Table 3). Households with and without LOE had no statistically significant difference in interpretation of positive test results (Table 2). Results of the propensity-weighted sensitivity analyses were consistent with the unweighted analysis (S2 Table).

**Table 2:** Adjusted odds ratio between language other than English (LOE) status and three primary outcomes: Vaccine intentions, positive COVID-19 test perceptions, and negative COVID-19 test perceptions.

	Outcomes^1^		
	Vaccine Intentions	Positive COVID-19 Test Perceptions	Negative COVID-19 Test Perceptions
	Adjusted OR (95%CI)	Adjusted OR (95%CI)	Adjusted OR (95%CI)
**Household Language Other Than English**	1.28 (0.74, 2.23)	1.27 (0.73, 2.22)	0.56 (0.34, 0.95)*
**Covariates**			
**Education** (Referent: GED or Some College)			
< 12^th^ grade	2.67 (0.80, 8.89)	0.38 (0.15, 0.97)*	0.71 (0.27, 1.89)
Bachelor’s Degree	1.11 (0.75, 1.64)	1.39 (0.94, 2.07)	1.18 (0.74, 1.87)
Advanced Degree	1.66 (1.01, 2.75)*	1.13 (0.72, 1.76)	1.18 (0.68, 2.06)
**Income** (Referent: $50,000 –$74,999)			
< $35,000	0.38 (0.21, 0.71)*	1.57 (0.81, 3.03)	1.08 (0.55, 2.12)
$35,000–$49,999	0.53 (0.27, 1.05)	1.29 (0.62, 2.69)	1.72 (0.75, 3.95)
$75,000–$99,999	0.84 (0.47, 1.49)	0.94 (0.53, 1.64)	1.23 (0.65, 2.30)
≥ $100,000	1.51 (0.87, 2.63)	1.12 (0.67, 1.87)	1.83 (1.02, 3.29)*
Not Reported	0.61 (0.33, 1.11)	0.88 (0.49, 1.58)	1.48 (0.75, 2.91)
**Child’s Health Insurance** (Referent: Private)			
Public Insurances^2^	1.20 (0.81, 1.79)	1.04 (0.71, 1.52)	1.18 (0.75, 1.87)
Other Insurances	0.57 (0.21, 1.55)	0.51 (0.21, 1.26)	0.46 (0.18, 1.2)
**Child Age** (Referent: ≥ 12 years old)			
< 12 years old	0.37 (0.27, 0.51)*	0.95 (0.70, 1.30)	0.91 (0.63, 1.32)
**Complex Chronic Conditions** (Referent: 1 CCC)			
≥ 2 CCCs	1.50 (1.03, 2.18)*	0.75 (0.53, 1.06)	1.81 (1.16, 2.85)*
**Expected likelihood of severe health effects due to COVID-19** (Referent: Not at all likely)			
A little likely, Somewhat likely	1.15 (0.81, 1.64)	1.80 (1.29, 2.50)*	1.55 (1.04, 2.31)*
Very likely, Extremely likely	2.23 (1.26, 3.94)*	1.98 (1.15, 3.41)*	0.88 (0.5, 1.54)
**Hospital Encounters in 2020** (Referent: 0)			
≥ 1 Hospital Encounter	0.83 (0.56, 1.25)	1.43 (0.93, 2.20)	0.85 (0.54, 1.36)
**High trust in the following sources for correct COVID-19 information** (Referent: Less trust)^3^			
Your doctor or healthcare provider	3.46 (2.34, 5.12)*	1.63 (1.07, 2.49)*	2.51 (1.58, 3.97)*
The US Coronavirus Task Force	7.66 (4.93, 11.89)*	1.40 (0.99, 1.96)	1.22 (0.81, 1.83)
Close friends and family members	0.74 (0.49, 1.13)	0.79 (0.52, 1.18)	0.69 (0.43, 1.09)

^1^Outcomes:

Vaccine Intentions = Child has received at least 1 COVID-19 vaccine or the caregiver responded “very likely” to the question, “How likely is your child to get an approved COVID-19 vaccine when it becomes available?”

Positive COVID-19 Test Perceptions = In response to “If my child gets a positive result, it means…”, caregiver selects “My child will need to isolate themselves from others” and “My child will not be able to attend school.”

Negative COVID-19 Test Perceptions = In response to “If my child gets a negative result, it means…”, caregiver selects “My child doesn’t have COVID-19 now” and does not select “My child can be around others without getting the virus from them.”

^2^The ’Public Insurances’ category includes participants with both public and private insurance. The ‘Other Insurances’ category includes participants who selected “Does not have health insurance”, “Don’t Know”, or “Prefer not to answer.”

^3^In response to “How much do you trust each of these sources to provide correct information about COVID 19 as it relates to your child?”, trust is dichotomized as High = “A great deal” and Less = “Not at all”, “A little”, “Somewhat”, and “Don’t Know”

* Significant Association

**Table 3:** Outcome frequencies for COVID-19 vaccine intentions, COVID-19 testing perceptions, and level of trust in various sources for COVID-19 information by language other than English (LOE) status.

	LOE n (col %)	Not LOE n (col %)	χ^2^ p-value
Total, *n (row %)*	133 (10)	1205 (90)	
**COVID-19 Vaccine Intention & Practices**			
Child is vaccinated or is very likely to be vaccinated for COVID-19^1^			
Yes	107 (80)	966 (80)	0.9376
No	26 (20)	239 (20)	
**COVID-19 Test Perceptions**			
If my child gets a **positive result**, it means:			
My child will need to isolate themselves from others	116 (87)	1046 (87)	0.8936
My child will not be able to attend school	126 (95)	1155 (96)	0.5461
If my child gets a **negative** test result, it means			
My child doesn’t have COVID-19 now	119 (89)	1118 (93)	0.1707
My child can be around others without getting the virus from them	16 (12)	33 (3)	< .0001*†
**Trust in Pandemic Information Sources** ^2^ ^,^ ^3^			
Your doctor or health care provider, n = 1338			
High	110 (83)	1046 (87)	0.1908
Low	23 (17)	159 (13)	
Your faith leader, n = 1293			
High	20 (15)	116 (10)	0.0616
Low	111 (85)	1046 (90)	
Your close friends and members of your family, n = 1338			
High	29 (22)	188 (16)	0.0655
Low	104 (78)	1017 (84)	
People at work/ class or other people you know, n = 1336			
High	17 (13)	133 (11)	0.5496
Low	116 (87)	1070 (89)	
Your contacts on social media, n = 1332			
High	3 (2)	31 (3)	1.0000†
Low	129 (98)	1169 (97)	
The US government, n = 1331			
High	47 (35)	312 (26)	0.0108*
Low	82 (64)	890 (74)	
The US Coronavirus Task Force, n = 1338			
High	62 (47)	568 (47)	0.9092
Low	71 (53)	637 (53)	

^1^Outcome is defined as the child has received at least 1 COVID-19 vaccine or the caregiver responded “very likely” to the question, “How likely is your child to get an approved COVID-19 vaccine when it becomes available?”

^2^In response to “How much do you trust each of these sources to provide correct information about COVID 19 as it relates to your child?”, trust is dichotomized as High = “A great deal” and Less = “Not at all”, “A little”, “Somewhat”, and “Don’t Know”

^3^Sample sizes for the trust variables may be smaller than the total sample size due to missing observations.

* Significant association

^†^ Fisher’s Exact Test

### Primary outcomes and covariate associations

In multivariable models, study outcomes were associated with several covariates (Table 2). Vaccine intentions were associated with higher education, older child age, more CCCs and greater perceived severity of illness from COVID-19. Notably, the largest effect sizes were observed with trusted sources being your doctor or healthcare provider (3.46 [2.34–5.12]) and the US Coronavirus Task Force (7.66 [4.93–11.89]). Expected interpretation of positive COVID-19 test results had similar associations as vaccine intentions, although they were less consistent and lower magnitude. Expected interpretation of negative COVID-19 test results was associated with higher income, more CCCs, and trusted source being ones doctor or healthcare provider.

### Secondary outcome and LOE status: COVID-19 sources of trust

Caregiver respondents from households with LOE were significantly more likely to trust the US government as a source of COVID-19 information as compared to those from non-LOE households (aOR [95% CI]: 1.86 [1.24–2.81]) (Table 4). However, most caregivers had low trust in the US government (64% LOE households and 74% non-LOE households reported absent trust). We observed no association between household LOE and trust in doctor/healthcare provider, faith leader, close friends/families, social media contacts or US Coronavirus Task Force. Similar levels of high trust in doctor or health care provider were reported by LOE and non-LOE caregivers (83% and 87%, respectively).

**Table 4:** Adjusted odds ratio between language other than English (LOE) status and a high level of trust in various sources for COVID-19 information.

	Trust in the following sources to provide correct information about COVID 19 as it relates to your child^1^						
	Doctor or Healthcare Provider	Faith Leader	Close Friends and Family	Work or School Colleagues	Social Media Contacts	U.S. Government	U.S. Coronavirus Task Force
	Adjusted OR (95%CI)	Adjusted OR (95%CI)	Adjusted OR (95%CI)	Adjusted OR (95%CI)	Adjusted OR (95%CI)	Adjusted OR (95%CI)	Adjusted OR (95%CI)
**Household Language Other Than English**	0.84 (0.50, 1.41)	1.32 (0.76, 2.29)	1.26 (0.78, 2.02)	1.02 (0.58, 1.81)	0.55 (0.15, 2.03)	1.86 (1.24, 2.81)*	1.14 (0.77, 1.69)
**Covariates**							
**Education** (Referent: GED or some college)							
< 12^th^ grade	2.46 (0.81, 7.47)	1.11 (0.39, 3.11)	1.41 (0.58, 3.40)	1.80 (0.63, 5.14)	2.03 (0.47, 8.75)	1.18 (0.42, 3.29)	1.18 (0.50, 2.76)
Bachelor’s degree	1.46 (0.98, 2.16)	0.75 (0.47, 1.19)	0.85 (0.58, 1.24)	0.84 (0.53, 1.33)	0.23 (0.07, 0.73)*	1.56 (1.10, 2.19)*	1.97 (1.47, 2.63)*
Advanced degree	2.23 (1.33, 3.75)*	0.59 (0.34, 1.02)	0.82 (0.53, 1.27)	1.14 (0.70, 1.86)	0.52 (0.18, 1.51)	2.35 (1.63, 3.39)*	3.13 (2.25, 4.34)*
**Income** (Referent: $50,000 –$74,999)							
< $35,000	1.26 (0.69, 2.30)	1.11 (0.57, 2.15)	1.45 (0.82, 2.56)	1.21 (0.61, 2.41)	1.87 (0.59, 5.94)	0.87 (0.49, 1.54)	0.84 (0.52, 1.36)
$35,000 –$49,999	1.36 (0.67, 2.73)	1.25 (0.61, 2.57)	1.17 (0.61, 2.23)	0.79 (0.34, 1.82)	0.65 (0.12, 3.52)	0.68 (0.36, 1.29)	0.63 (0.37, 1.08)
$75,000 –$99,999	1.05 (0.59, 1.85)	0.62 (0.30, 1.27)	0.72 (0.40, 1.29)	0.95 (0.50, 1.79)	0.85 (0.22, 3.35)	0.92 (0.57, 1.49)	0.74 (0.48, 1.12)
≥ $100,000	1.79 (1.04, 3.07)*	0.78 (0.43, 1.41)	0.94 (0.58, 1.55)	0.80 (0.45, 1.40)	0.79 (0.23, 2.69)	1.26 (0.83, 1.91)	1.04 (0.72, 1.52)
Not reported	0.69 (0.39, 1.21)	0.91(0.46, 1.83)	0.71 (0.38, 1.33)	0.83 (0.41, 1.68)	0.64 (0.14, 2.88)	0.49 (0.27, 0.87)*	0.47 (0.29, 0.75)*
**Child’s Health Insurance** (Referent: Private)							
Public Insurances^2^	0.63 (0.42, 0.93)*	1.25 (0.80, 1.96)	1.08 (0.74, 1.57)	0.83 (0.54, 1.29)	1.07 (0.43, 2.65)	1.00 (0.73, 1.38)	0.86 (0.65, 1.14)
Other Insurances	0.21 (0.09, 0.52)*	1.88 (0.65, 5.47)	2.91 (1.24, 6.83)*	1.26 (0.41, 3.91)	2.22 (0.36, 13.65)	1.17 (0.45, 3.05)	0.80 (0.34, 1.85)
**Child Age** (Referent: ≥ 12 years old)							
< 12 years old	1.11 (0.79, 1.55)	1.41 (0.98, 2.02)	0.98 (0.72, 1.33)	1.39 (0.98, 1.96)	0.63 (0.29, 1.35)	1.00 (0.78, 1.30)	0.93 (0.74, 1.18)
**Complex Chronic Conditions** (Referent: 1 CCC)							
≥ 2 CCCs	0.89 (0.62, 1.27)	0.97 (0.64, 1.46)	1.09 (0.78, 1.52)	1.12 (0.76, 1.65)	0.75 (0.33, 1.70)	0.85 (0.63, 1.13)	1.08 (0.83, 1.40)
**Expected likelihood of severe health effects due to COVID-19** (Referent: Not at all likely)							
A little likely, Somewhat likely	1.8 (1.26, 2.58)*	1.08 (0.71, 1.64)	1.18 (0.85, 1.66)	1.15 (0.78, 1.68)	0.91 (0.41, 2.00)	0.99 (0.75, 1.30)	1.08 (0.84, 1.38)
Very likely, Extremely likely	1.58 (0.94, 2.68)	1.06 (0.58, 1.92)	0.91 (0.54, 1.53)	0.83 (0.45, 1.54)	0.99 (0.33, 2.95)	0.98 (0.63, 1.53)	1.37 (0.93, 2.03)
**Hospital Encounters in 2020** (Referent: 0)							
≥ 1 Hospital encounter	0.88 (0.58, 1.34)	1.46 (0.94, 2.27)	1.18 (0.81, 1.73)	1.02 (0.65, 1.60)	1.04 (0.43, 2.51)	1.19 (0.86, 1.66)	0.84 (0.62, 1.14)

^1^In response to “How much do you trust each of these sources to provide correct information about COVID 19 as it relates to your child?”, trust is dichotomized as High = “A great deal” and Less = “Not at all”, “A little”, “Somewhat”, and “Don’t Know”

^2^The ’Public Insurances’ category includes participants with both public and private insurance. The ‘Other Insurances’ category includes participants who selected “Does not have health insurance”, “Don’t Know”, or “Prefer not to answer.”

*Significant Association

## Discussion

This cross-sectional survey of caregivers from Spring 2022 observed high vaccine intentions in households of CMC with and without LOE; however, interpretations of COVID-19 test results and trusted information sources varied. As national pandemic attention shifts towards living with COVID-19, vaccine and test perceptions have important implications due to their central role in ongoing mitigation. Because differences in vaccine and test perceptions may contribute to health disparities, vulnerable populations deserve ongoing attention. In our study, the combination of both medical complexity and household LOE likely represents a uniquely vulnerable population. Our hypothesis that LOE status would have associations with vaccine and test perceptions that may promote disparities in COVID-19 outcomes was partially confirmed. A concerning observation is that households with LOE more often overinterpreted their child’s safety in the community following a negative test.

Family awareness of their child’s vulnerability may explain why vaccine intentions in our respondents (80%) were much higher than the general US child population, where only 28% of parents reported high vaccine intentions [33]. Our results also differ from studies in which LOE was associated with lower vaccine intentions and uptake [15, 36]. We speculate that CMC’s health vulnerabilities may explain uniformly higher vaccination rates for CMC, even amongst linguistically diverse populations. Families may be motivated by their child’s higher risk of hospitalization and severe COVID-19 [8, 21, 25], as well as greater consequences from pandemic public health measures that can reduce health care access [37, 38]. Alternatively, as we did not have a measure of English proficiency, it is possible that disparities may have been observed if language proficiency rather than household language had been assessed [39]. In addition, vaccine intentions may be linked to sociodemographic and cultural factors not accounted for in our analysis [40, 41].

Although vaccine intentions of caregivers increased from 65% in Summer 2021 [32] to 80% in Spring 2022, opportunities remain to improve vaccination rates. Despite continually updated vaccine recommendations with contemporary vaccine formulations, COVID-19 vaccine hesitancy exists [42, 43] and the majority of children are not currently up to date [44]. It is plausible that vaccine intentions have shifted amidst booster recommendations, and researchers should explore whether households with LOE have developed disparities as this population may require distinct solutions [45].

In contrast to high vaccination intentions at the time of the study, interpretation of COVID-19 test results illustrated more variability by household language. COVID-19 testing will likely remain an important mitigation tool for the foreseeable future, and growing reliance on at-home testing places greater importance on family interpretation of results [46]. Conducting and interpreting at-home tests is a complicated process [47]. It is plausible that testing procedures are even more challenging for individuals with LOE. For example, families with LOE are known to experience comprehension challenges in medical encounters and reading prescription medication labels [48].

Our results suggest that in households with LOE, families of CMC report important differences in interpretation of COVID-19 test results for negative results. Interpreting negative COVID-19 tests is particularly important considering the known limitations of antigen test performance [49]. In fact, CMC families have expressed concern that a negative test may provide a false sense of security for their high-risk child [50]. This observation is consistent with our results, i.e., households with LOE may be over-interpreting their child’s safety after a negative test since they more often perceived that their child can be around others without getting the virus from them after a negative test. It is conceivable that such differences may contribute to disparities in COVID-19 outcomes.

To influence public health pandemic knowledge and behaviors, messaging through trusted sources is important, and trusted information sources appear to differ across populations. We were surprised to observe that households with LOE reported higher trust in the government than non-LOE households. Other research evaluating trust in the government for COVID-19 information is mixed, with some studies demonstrating overall low trust in the White House for COVID-19 information [51] and others finding governmental websites as the most trusted source of information [52]. Our survey did not distinguish between local public health departments, state and federal governmental agencies. Delineating this in future studies may identify the most trusted sources for LOE families. Although trust in the government was higher among households with LOE, it was still relatively low overall, and exploring other avenues of COVID-19 messaging is warranted.

The highest level of caregiver trust for COVID-19 information was seen in health care providers. Given the absence of high trust in other sources queried, the duty and stakes are high for clinicians to deliver accurate information effectively, including for linguistically and culturally diverse populations [2, 4]. Trust in health care providers has been linked to increased vaccine uptake [33, 53, 54], with parents reporting increased likelihood of COVID-19 vaccination if their child’s healthcare provider recommended it [55]. Given the high frequency of health care encounters among CMC [56], opportunities exist for public health education during clinical care. In fact, CMC assisted by technology (e.g., ventilation via tracheostomy) were more likely to receive the COVID-19 vaccine when counseled by primary care provider or subspecialist [57].

Finally, despite observing relatively few disparities in our analysis, this topic requires continued focus. Navigating the complexity of changing COVID-19 public health recommendations and vaccine schedules is likely an obstacle for LOE families [58, 59]. Prior studies have found COVID-19 media are typically provided in only a few languages and people speaking LOE have noted inconsistent and sometimes discriminatory messages [60, 61]. Providing educational material with culturally appropriate terminology in the patient’s language is one way of improving equitable access to COVID-19 information [62]. In addition, studies have demonstrated that even when COVID-19 information is communicated in a non-English language, the information provided is often inferior [63]. Provider education on effective communication and language service use [64, 65] along with further research dedicated to delineating best practices in communicating COVID-19 information to people who speak language other than English is needed.

### Limitations

Our study is subject to several limitations. This is a single-center study in the Midwest; therefore, results may not generalize to other linguistically diverse regions and populations. While the proportion of LOE households in our study is similar to our region [66], the survey response rate was 49% and suggests the respondent sample likely does not represent all LOE households in our region. The small sample size of LOE households reduces the power of the study to explore more nuanced sub-analyses, such as whether the results differed based on specific LOE. The survey was only available in English and Spanish. Results may have differed if we had offered the survey in additional languages, as some questions such as those regarding test perceptions may be confusing when translated into different languages. All non-English languages were evaluated together, and due to limited power and the large number of additional languages, we were unable to assess for language-related associations connected to social norms, cultural and political influences, etc. We cannot account for potential social desirability bias in family-reported pandemic perceptions and possible selection bias of caregivers more engaged in healthcare being more likely to complete the survey. Our study assessed families with household language other than English, but did not assess English language proficiency or multilingual households. Finally, the measures were family-reported and standardized across all RADx-UP programs. We could not confirm vaccine histories or distinguish antigen or polymerase chain reaction COVID-19 test perceptions.

## Conclusion

This cross-sectional survey of CMC caregivers demonstrated high COVID-19 vaccine intentions for households with and without LOE. However, households with LOE interpreted negative COVID-19 tests in ways that overestimated child safety compared to households with LOE. Future research should explore whether such differences contribute to disparities in COVID-19 outcomes. Since sources of trust for pandemic information differed based on household language, targeted public health messaging strategies should leverage the most trusted sources for specific populations. Addressing language-based disparities in culturally appropriate ways is critical to the COVID-19 and future pandemic response.

## Supporting information

S1 Table Caregiver and child demographic characteristics by response status, *n* = 3080. (DOCX)

S2 Table Adjusted odds ratio of primary outcomes with and without propensity score inverse probability weighting for likelihood of survey response. (DOCX)

## Abbreviations

aOR: Adjusted odds ratio
CCC: Complex chronic condition
CI: Confidence interval
CMC: Children with medical complexity
COVID-19: Coronavirus disease 2019
LOE: Language other than English
RADx-UP: Rapid Acceleration of Diagnostics-Underserved Populations
STROBE: Strengthening the Reporting of Observational Studies in Epidemiology
US: United States
