# Supplementary material for: Disparities in COVID-19 vaccine intentions, testing and trusted sources by household language for children with medical complexity
Source: PLoS One. 2024 Jun 14;19(6):e0305553. doi: 10.1371/journal.pone.0305553 (PMC11178204; doi:10.1371/journal.pone.0305553)
Supplement: S2 Table — (DOCX) [file pone.0305553.s002.docx]

**Supporting information Table 2.** **Adjusted Odds Ratio of Primary Outcomes with and without Propensity Score Inverse Probability Weighting for Likelihood of Survey Response.**

|  | Primary Analysis | Inverse Probability Weighted^1^ Sensitivity Analysis |
| --- | --- | --- |
|  | aOR (95% CI) | aOR (95% CI) |
| Vaccine Intentions | 1.28 (0.74, 2.23) | 1.05 (0.72, 1.54) |
| Positive COVID-19 Test Perceptions | 1.27 (0.73, 2.22) | 1.32 (0.87, 2.01) |
| Negative COVID-19 Test Perceptions | 0.56 (0.34, 0.95)* | 0.64 (0.43, 0.96)* |
| ^1^Propensity score weights assess the likelihood of response, accounting for race/ethnicity, insurance type, child sex, child age, primary language, history of covid infection, number of CCCs, and number of hospital encounters in 2020.  *Statistically Significant Association | | |
